# Usage of unguided, guided, and blended care for depression offered in routine clinical care: Lessons learned

**DOI:** 10.1016/j.invent.2023.100670

**Published:** 2023-09-19

**Authors:** Fien Buelens, Patrick Luyten, Herwig Claeys, Eva Van Assche, Tom Van Daele

**Affiliations:** a Expertise Unit Psychology, Technology & Society, Thomas More University of Applied Sciences, Campus Sanderus, Molenstraat 8, 2018 Antwerp, Belgium; b Faculty of Psychology and Educational Sciences, KU Leuven, Tiensestraat 102 pobox 3722, 3000 Leuven, Belgium; c Research Department of Clinical, Educational and Health Psychology, University College London, 1–19 Torrington Place, London WC1H 7BG, United Kingdom; d Project manager OnlinePsyHulp associated with Zorggroep Zin, Salvatorstraat 25, 3500 Hasselt, Belgium

**Keywords:** Dropout, Online intervention, Guidance, Depressive symptoms, Self-help, Blended treatment

## Abstract

**Introduction:**

Internet-delivered psychotherapy is often considered to be a promising way to extend mental healthcare services around the world. Research findings that have emerged over the past two decades have strengthened this claim. However, very little is known about the usage of internet-delivered psychotherapy in real-life circumstances.

**Methods:**

The current study explored the real-life usage of depressiehulp.be, a publicly available online platform for depression that offers pure self-help, online guided self-help, and blended treatment for depression in Flanders, Belgium, using data collected from 2656 participants between May 2018 and May 2022.

**Results:**

Both duration of engagement with the online platform and number of exercises completed increased with increasing levels of therapist guidance. Findings also showed a particular pattern of engagement for each of the online treatments. Overall, participants completed most exercises during the first days of treatment. However, participants using pure online self-help showed the fastest decrease of engagement over time, with most dropping out after completing a few exercises, and more than half of all participants who enrolled in the self-help programme did not even begin the programme. In both guided and blended treatment, participants tended to show higher levels of engagement with the online platform. In each treatment modality, a relatively small but notable group of participants showed high levels of engagement. There was no relationship between severity of depression and duration of engagement.

**Conclusions:**

The current study demonstrates the importance of therapist support in online interventions and offers additional insights into how, and to what extent, online platforms are used. Future research should explore clinical impact and policy implications.

## Highlights

• A public online platform for depression offers insights into real-life use.
• Duration of engagement and number of exercises increase with more guidance.
• Most exercises are completed during the first days of treatment.
• Pure online self help shows the fastest decrease of engagement.

## 1 Introduction

“No physical health without mental health” was a phrase once coined by the World Health Organization and has become increasingly popular over the past decades (Kolappa et al., 2013). Although the importance of this statement is increasingly acknowledged, there are still several barriers to the effective delivery of mental health care. Common barriers include stigma and long waiting lists (Corrigan, 2004; Rens et al., 2021), resulting in high unmet need, which can be defined as the failure to provide necessary care to individuals struggling with mental health problems (Sherbourne et al., 2001).

Unmet needs are often considered in discussion of the so-called treatment gap (Pathare et al., 2018); this is the difference between the total number of individuals with a disorder and the number of individuals with that disorder who receive treatment (Kohn et al., 2004). In Europe, the treatment gap for major depression was estimated to affect up to 45 % of the people with major depression between 2000 and 2019 (Moitra et al., 2022), meaning that many individuals did not seek and/or receive appropriate treatment.

Many attempts have been made to address the treatment gap and help overcome these unmet needs. Digital mental health approaches in general, and internet-delivered psychotherapy in particular, are often considered to play an important role in addressing the treatment gap (Kazdin and Blase, 2011; Kazdin, 2019). Digital mental health interventions have the potential to reach currently hard-to-reach populations because they can be accessed directly and anonymously; they also have the potential to increase the effectiveness of traditional face-to-face psychotherapy, for example, through blended treatment (the combination of face-to-face consultations with technological means such as apps or virtual reality (Smith et al., 2023). Meta-analyses have demonstrated the effectiveness of digital mental health approaches (Andersson et al., 2019; Grist et al., 2019; Hilty et al., 2016; Jonsson et al., 2023).

However, online interventions have their own challenges. Studies have often found low levels of adherence to treatment, even in the context of randomized controlled trials (RCTs) (Ebert et al., 2018). Furthermore, different meta-analyses have reported high dropout rates (Andersson et al., 2019; Grist et al., 2019; Jonsson et al., 2023). For example, in a meta-analysis of 70 RCTs of smartphone-delivered self-help interventions, 24 % of the participants stopped using the app after 8 weeks, while 36 % of the participants had stopped using the app at the 12-week follow-up (Linardon and Fuller-Tyszkiewicz, 2019).

Similar and often worse results have been obtained in studies of internet-delivered treatments outside the context of RCTs. Baumel et al. (2019), for instance, found that only 3 % of participants still opened a self-help mental health application 1 month after installing it. Bendelin et al. (2011), in a qualitative study of participants who participated in an RCT of internet-delivered therapy, found that only a small proportion of users of a digital mental health application could be described as *doers*, who worked with the material in a practical way and applies it to real-life settings. Most of the participants were classified as *strivers*, who reported that they intended to use the online materials but often struggled to do so, or as *readers*, that is, participants who only read through the online materials and largely failed to apply them to their own problems.

In general, very little is known about the usage of online interventions in routine practice outside the confines of research studies (Van Daele et al., 2020), although there is increasing consensus that guided online interventions (e.g., when participants are supported by receiving personalized texts, automated e-mails, chat messages, or other forms of therapist support) achieve higher levels of adherence (Castro et al., 2018; Renfrew et al., 2020) and effectiveness compared with unguided self-help (Baumeister et al., 2014; Ciuca et al., 2018; Kass et al., 2014; Moskowitz et al., 2021). However, more research in this area is needed, particularly as very few studies have explored in detail usage data for guided and unguided self-help interventions in routine clinical care (Kemmeren et al., 2019; Kooistra et al., 2016). A meta-analysis focusing on guided self-help for depression and anxiety, for instance, reported mixed findings in this regard, as guided self-help was associated with a significantly greater reduction in symptoms and lower non-usage attrition compared with unguided self-help in only four out of nine included RCTs (Shim et al., 2017).

This study aimed to explore participants' use of a free, government-funded and publicly available online platform for depression in Flanders, Belgium, offering pure self-help, online guided self-help, and blended treatment, to investigate whether findings from research studies can be generalized to routine clinical care. Specifically, we investigated the following usage data across the three treatment modalities: the overall duration of engagement with the platform, the usage of specific modules, the evolution of usage over time, and the relationship between severity of depression and engagement.

First, consistent with findings in clinical trials (Baumeister et al., 2014; Ciuca et al., 2018; Kass et al., 2014; Moskowitz et al., 2021), we expected to find positive associations between therapist support, engagement with the platform, and participant usage. Severity of depression was expected to be negatively related to engagement. Our second set of hypotheses was related to usage over time, which was expected to decrease over time, but this decrease was expected to be more gradual in the treatment modalities involving more guidance (i.e., online guided self-help and blended treatment) compared with unguided self-help.

## 2 Methods

### 2.1 Sample

We retrieved data from all users who engaged with the platform over 4 years for which data were available (May 2018 to May 2022) (*n* = 2656 participants). Overall, 678 participants identified as male, 1948 as female, and 30 as non-binary.

### 2.2 Online platform

Depressiehulp.be is a free, government-funded, publicly available online platform serving approximately 6.6 million inhabitants in Flanders, the Flemish-speaking northern part of Belgium. It was first launched in 2017. The platform offers different online modules for depressive symptoms. Participants can follow three different treatment modalities: (1) self-help with automatic reminder e-mails, (2) online guided self-help, involving up to 12 chat sessions with a mental health professional over a period of 3 months, or (3) blended treatment in which online work is combined with face-to-face sessions with a mental health professional. Patients who participated in the self-help and online treatment were all self-referred as the platform is freely available for all inhabitants in the Flanders region of Belgium. Patients in blended treatment were referred by mental health professionals, as this treatment modality is currently only available in one of 20 publicly funded mental health centres in Flanders upon referral. The mental health professionals responsible for guided self-help and blended treatment are certified clinical psychologists and all had considerable training and supervision related to both the content and technical aspects of the online platform. They are also offered regular supervision and additional workshops after training as they are expected to deliver online treatment as part of their routine clinical practice.

In terms of content, both guided self-help and blended treatment entail a maximum of 17 modules. Mental health professionals can either select a predetermined set of modules based on evidence-based packages for depression (e.g., cognitive-behavioural therapy (CBT) or dynamic interpersonal therapy (DIT) for depression), but they can also tailor their trajectories to meet the individual needs of participants (e.g., they may add a module on relaxation techniques or mindfulness to each of the chosen trajectories). Consequently, the usage data in these two treatment modalities may be influenced by the preferences of the mental health professionals and needs of patients. The modules encompass (a) psychoeducation about depression, (b) modules based on evidence-based empirical approaches to depression treatment, such as CBT (behavioural activation, second-wave CBT, and third-wave CBT approaches such as acceptance and commitment therapy (ACT) and mindfulness), (c) psychodynamic therapy, namely, DIT, and (d) modules focusing on relaxation. Unguided self-help comprises 6 shortened versions of these modules, and participants have the freedom to complete as many modules as they wish and may determine the order in which they complete them. A full overview of all modules across the three treatment modalities can be found in Table 1.

**Table 1:** Overview of the online platform's modules and their content across the three treatment modalities.

Common modules	Behavioural activation	CBT	ACT	DIT
Self-help treatment modality				
- Mood barometer - Weekly planner - Library - Patient forum	- Activities - Ranking - Weekly planner - Stumbling blocks	- ABCDE - Automatic thoughts - Helpful thinking - Behavioural experiments - Relaxation	- Values - Goals - Action plan	- Relationship style - Self-image - Image of others - My relationship pattern - Advantages and disadvantages - Change

Guided self-help and blended treatment modalities				
- Mood barometer - Weekly planner - Symptoms - Strengths - My story - Psychoeducation - Relaxation - Rumination - Library - Forum	- Values - Activities - Ranking - Weekly planner - Stumbling blocks	- Observing vs. interpreting - Automatic thoughts - Helpful thinking - Behavioural experiments	- Values - Goals - Action plan - Mindfulness	- My relationships - Relationship style - Self-image - Image of others - Relations and emotions - My relationship pattern - Pattern reflecting - Advantages and disadvantages - Change

In terms of timing, the self-help treatment is typically recommended to be completed within a timeframe of 10 to 12 weeks, although patients are free to continue using the platform for as long as they wish. For the online guided self-help, the support provided by a mental health professional is limited to a period of 3 months or 12 sessions. Afterwards, patients can continue using the platform without professional guidance. For the blended treatment, in the Belgium mental health care system there is no limit in terms of the number of therapy sessions offered in government-funded mental health centres, but therapists are expected to evaluate their treatment at least every 6 months and decide, jointly with the patient, whether additional treatment is needed. Treatment length for most patients ranges between 6 and 18 months. A future research report will focus on the ratio between face-to-face sessions and online work in blended treatment offered through depressiehulp.be.

Finally, participants are asked to complete the Patient Health Questionnaire (PHQ-9, Kroenke et al., 2001) as part of the routine screening before the start of each program. Participants could complete the PHQ-9 questionnaire repeatedly. However, the majority of the participants (71 %) completed the PHQ-9 only once over the time of the treatment. Based on this finding, we decided to use the PHQ-9 data collected before the start of the treatments and defined this as PHQ-9 at baseline.

### 2.3 Data analysis

Data on participant usage were retrieved for 2656 participants over 4 years for which complete data were available (May 2018 to May 2022). For data analytic purposes, we had to make a number of assumptions. First, if a participant had saved a particular exercise and then saved the same exercise again within 1 h, it was considered as a continuation of the previous exercise and not as a separate, new exercise. A second assumption was made regarding the total duration of engagement, which was calculated as the difference in days between the first and the last exercise within a trajectory. A trajectory was defined as the period between the first time and the last time a participant was active on the platform. However, if a participant was inactive for at least 3 months and then started using the online platform again, this was considered as a new trajectory. Furthermore, we aimed to consider only ‘completed’ trajectories and to exclude participants who were still using the platform. Therefore, we excluded all participants who were still active on the platform in the 3 months before the end of the data-collecting period. Finally, all participants who were active on the online platform for only 1 day were considered to be early dropouts and were not included in the analyses. The raw original data consisted of 2656 participants and 2846 trajectories. In total, 1671 (59 %) trajectories consisted of early dropouts: 1225 in self-help, 312 in online guided self-help, and 134 in blended treatment. These early dropouts were not considered in subsequent analyses, which focused on the 1175 trajectories lasting >1 day. Due to the lack of data, the analyses the PHQ-9 are based on 892 trajectories.

R (R Core Team, 2021) was used for data processing. The duration of engagement and number of exercises within a trajectory were investigated with non-parametric tests, as the assumptions for parametric tests were violated. SPSS version 28 (IBM Corp, 2021) was used to conduct a descriptive analysis, alongside a Kruskal–Wallis test and Dunn's pairwise test. Pearson product-moment correlations were calculated between baseline severity of depression as assessed with the PHQ-9 and total duration of engagement. To investigate the evolution of the total number of exercises and the number of exercises saved in the most frequently used modules over time, descriptive analyses were conducted in SPSS. All graphs were made by using the R package ggplot2 (Wickham, 2016). The number of exercises in the most frequently used modules over time was calculated by adding the frequencies of attempts of the different exercises (defined as engagement with an exercise that was subsequently saved by the participant) within a module.

## 3 Results and discussion

### 3.1 Total duration of engagement

**Fig. 1:**
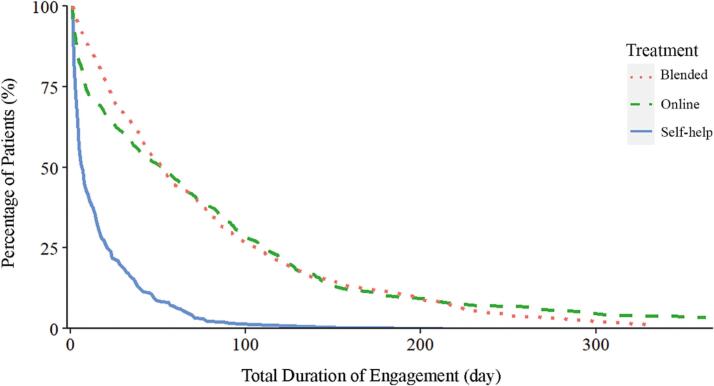
Engagement with the platform across the three different treatment modalities.

A Kruskal–Wallis test showed that the different treatment modalities differed significantly in the total duration of engagement (Table 2). Furthermore, Dunn's pairwise tests showed a significant difference between the treatment modalities. The total duration of engagement was significantly longer in the online guided self-help and blended modalities compared with the pure self-help modality (*p* < .001). Moreover, the total duration of engagement was significantly longer in the online guided self-help compared with the blended treatment (*p* = .045). Fig. 1 shows the engagement with the online platform over time in terms of the percentage of participants in each of the three modalities.

**Table 2:** Descriptive statistics and results of a Kruskal–Wallis test for the three different treatment modalities.

	Self-help	Online guided self-help	Blended treatment	*χ*^*2*^	*df*	*p*
Total duration of engagement (number of days)						
*n*	308	483	384			
*M*	16.33	80.72	79.46			
Mean rank	329.37	659.27	705.80	246.73	2	<0.001

Usage (number of exercises)						
*n*	308	483	384			
*M*	11.92	34.15	30.76			
Mean rank	442.68	624.25	658.96	78.90	2	<0.001

Participants scored on average 15.26 on the PHQ-9, indicative of moderate to severe depression (Kroenke et al., 2001). A Kruskal–Wallis test showed that the different treatment modalities differed significantly in baseline severity of depression (Table 3). A Dunn's pairwise test showed significance differences between the self-help and online treatment (*p* < .001) and between the online and blended treatment (*p* < .001). The patients in the online treatment modality scored higher on the PHQ-9 than the patients in the self-help and online treatment. The self-help and blended treatment showed no difference in depression scores (*p* = .66). There was no significant correlation between baseline severity of depression scores and the total duration of engagement across all treatment modalities: *r*(892) = −0.031, *p* = .362. Similarly, non-significant correlations or small correlations were found between baseline severity of depression and engagement in the self-help, online and blended treatment modalities, *r*(296) = −0.117, *p* = .044; *r*(440) = −0.147, *p* = .002 and *r*(156) = −0.061, *p* = .362, respectively.

**Table 3:** Descriptive statistics and results of a Kruskal–Wallis test for the PHQ-9.

	Self-help	Online guided self-help	Blended treatment	*χ*^*2*^	*df*	*p*
PHQ-9 before treatment						
*n*	296	440	156			
*M*	14.12	16.70	13.34			
Range	2–27	0–27	0–25			
Mean rank	392.15	513.42	360.88	60.25	2	<0.001

Importantly, although a sizeable group of participants dropped out early, there was also a group of participants who remained engaged with the platform for prolonged periods of time. Over time, the number of these participants gradually decreased, with the highest number of participants remaining active in online guidance the longest (Table 4).

**Table 4:** Number of participants for the three different treatment modalities engaging with the platform for at least 6 months, compared to the total number of trajectories (*N* = 1175).

Months after starting	Across the three modalities		Self-help		Online guided		Blended	
	n	%	n	%	n	%	n	%
6	93	7.91	1	0.08	49	4.17	43	3.66
9	43	3.66	0	0	29	2.47	14	1.19
12	22	1.87	0	0	17	1.45	5	0.42

### 3.2 Usage

The three treatment modalities differed significantly in the numbers of exercises attempted, according to a Kruskal–Wallis test (Table 1). Dunn's pairwise tests showed that significantly more exercises were attempted in the online and blended modalities compared with the self-help modalities (*p* < .001), with more exercises being attempted in the online guided self-help modality compared with the blended modality (*p* = .403).

There was no significant correlation between the number of exercises and the baseline severity of depression scores across all treatment modules: *r*(892) = −0.024, *p* = .473. Furthermore, significant, but small, negative correlations were found between baseline severity of depression and number of exercises in the self-help and online treatment, but not in the blended treatment modalities, *r*(296) = −0.123, *p* = .034; *r*(440) = −0.129, *p* = .007 and *r*(156) = 0.062, *p* = .445, respectively.

### 3.3 Evolution of usage over time

**Fig. 2:**
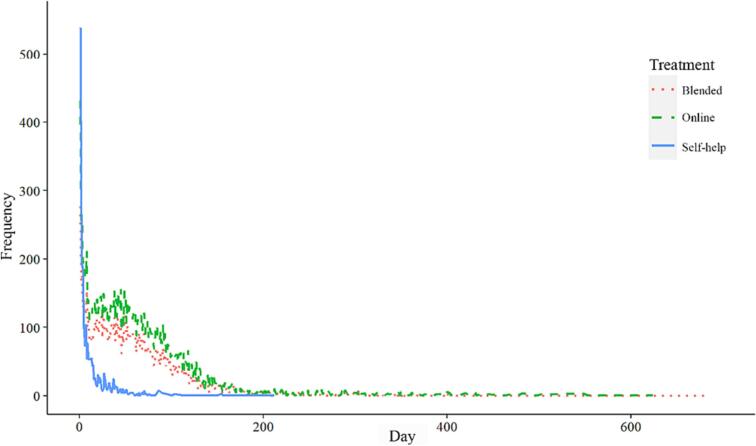
Number of exercises in the trajectories over time in the three different treatment modalities.

Participants completed most exercises in the first days after enrolling in the programme. In unguided self-help, there was an early peak in the number of exercises completed followed by a very rapid decline for most participants (Fig. 2). Participants completed about half of the exercises (51 %) within the first 5 days after enrolment (Table 5). Similarly, in both the online guided and blended modalities, there was a peak at the start of the treatment period in terms of the number of exercises completed, but this peak was much less pronounced compared with that seen in unguided self-help. In online guided self-help and blended treatment, 9 % and 8 %, respectively, of the exercises were attempted in the first 5 days. Moreover, the decrease over time in these treatment modalities was much less pronounced than that in unguided self-help.

**Table 5:** Percentage of exercises attempted over time in the three treatment modalities.

	After 5 days	After 7 days	After 30 days	After 60 days	After 90 days
Self-help	51 %	58 %	87 %	97 %	99 %
Online	9 %	11 %	31 %	56 %	75 %
Blended	8 %	11 %	32 %	59 %	77 %

Furthermore, in the online guided self-help condition half of the exercises were attempted within 53 days, very similar to what was observed in blended treatment (50 days). Both trajectories also had a slight increase in usage around day 50, which is most likely explained by the fact that by that time most participants had entered the middle phase of treatment, which typically involved modules focusing on fostering change in habitual patterns of feeling, thinking, and behaving.

### 3.4 Usage of the different modules

The most frequently used module (60.3 % of the saved exercises) was the ‘Mood Barometer’, which is available by default for all participants and essentially allows participants to track their mood across time. The second most frequently used module was ‘Helpful Thinking’ (5.3 % of saved exercises). This module is rooted in cognitive-behavioural approaches to depression and focuses on dysfunctional thoughts and assumptions, and strategies to challenge automatic thoughts and assumptions. The third most frequently used module was ‘My Thoughts’ (4.7 %). This module, which is again rooted in cognitive-behavioural approaches to depression, focuses on relationships between thoughts, feelings, and actions, that is, the cornerstone of second-wave approaches to depression. The fourth most used module was ‘Relationship Patterns’ (4.7 %), which was developed based on psychodynamic and interpersonal approaches to the treatment of depression. In this module, participants are invited to elaborate on their typical attachment and interpersonal style and its possible relationships with the onset and course of their depressive symptoms. Exercises within this module also focus on the advantages and disadvantages of this pattern of relating to self and others. The fifth most used module was ‘Week Planner’ (3.1 %), which can be used by participants to help structure their daily activities.

**Fig. 3:**
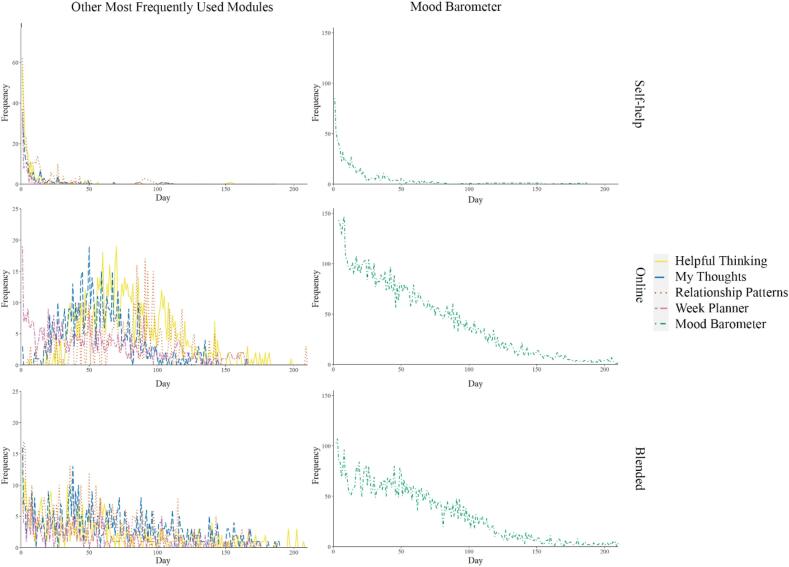
Number of exercises in the most frequently used modules over the time in different treatment modalities. *Note*. The axes of the figures are different for better visibility.

Fig. 3 shows the change in the number of saved exercises in the five most used modules across the self-help (2487 exercises), online guided self-help (15,038 exercises), and blended (10,674 exercises) treatment modalities. Visual inspection showed that ‘Mood Barometer’ clearly had the highest frequency of use in all three treatment modalities, with few differences among modalities but the steepest decline for the self-help modality. All other modules were used less and their use also showed a more gradual decline, except in the online guided self-help modality, which showed a slight increase in the number of exercises attempted between day 50 and day 100.

### 3.5 Discussion

Online platforms are increasingly considered as a means to facilitate the delivery of mental health services. Although dropout and poor adherence nevertheless seem to reduce their potential impact, little research to date has focused on how participants interact with these platforms in real life, outside the context of research studies. This is one of the first studies to report usage data based on a publicly available platform.

Results showed that participants who received guidance engaged with the online platform for longer, with the blended treatment being associated with the longest engagement. These results confirm findings from other studies (Castro et al., 2018) indicating that adherence to online psychological help tends to increase with additional support. The high levels of early dropout and the steep decline in usage in the self-help modality also reflect earlier findings regarding high dropout and low adherence in non-selected online self-help interventions (Baumel et al., 2019; Linardon and Fuller-Tyszkiewicz, 2019). A similar more gradual but relatively continuous decline in usage of guided self-help and blended treatment modalities was previously reported by Renfrew et al. (2020), who found that adherence tended to increase with additional support beyond self-help alone. The current study found that participants not only engaged for longer but also engaged more when guidance was provided: they attempted more exercises, irrespective of the type of guidance provided, although most exercises were attempted in the first days of the trajectories. A small but important subgroup of participants showed persistent and considerable engagement with the platform; this group may match the subgroup of ‘doers’ identified in an earlier qualitative study of the use of online help for psychological problems (Bendelin et al., 2011). Finally, participants engaged primarily with a relatively small number of modules. Further research is needed to investigate potential dose–response relationships between the number of modules and exercises completed by participants and outcomes.

The results of this study have important implications for users, practitioners, researchers, and policymakers. First, although there seems to be a small proportion of people who can engage with unguided online self-help for depression, most participants seem to benefit from guidance provided by a mental health professional, at least in terms of increasing their engagement in treatment. Second, adherence to treatment in the blended treatment and the online guided self-help modalities was very similar, and thus it is as yet unclear whether in-person face-to-face contact is required to achieve optimal adherence. There may be substantial differences in the nature of the participants, but not in baseline severity of depression scores, who enrolled in the guided self-help and blended treatment modalities in this study, and further research in this area is needed. Third, the number of modules used by participants in the online self-help modality was relatively small. In the absence of effectiveness data, it is difficult to interpret these findings. Yet, in traditional face-to-face psychotherapy, there is similarly no consensus regarding the optimal amount of sessions and duration of treatment for depression (Falkenström et al., 2016). Consequently, this factor seems important to further explore in future research.

This leads us to discuss a number of important limitations of this study. First, because no data were available concerning the effectiveness of the treatment offered as there were no post-depression scores, we could not investigate potential dose–response relationships or differences in the effectiveness of the different treatment modalities. Currently, the online platform does collect outcome data, so it is hoped that future work will be able to answer these questions. Second, and similarly, because participants in the unguided self-help and guided self-help modalities are able to receive help anonymously, we could not compare features (e.g., demographic and clinical features) of participants in these two groups with participants in the blended treatment. For instance, it would be interesting to investigate the demographic and clinical features of the small group of participants with a high level of engagement. Finally, the results of this study may not generalize to online interventions for other types of psychopathology and to the engagement of participants with online help for depression in other countries.

## 4 Conclusion

In conclusion, the current study is one of the first to offer insights into how an online platform for depressive symptoms is being used in real life and how the extent of guidance provided by a mental health professional relates to the way and extent the platform is subsequently used. More research is required to gain further insights and should definitely be pursued, as online interventions are promising tools to address unmet needs in mental health care.

## Funding statement

No specific funding. Research was performed as part of the employment of the authors.

## Declaration of competing interest

PL and HC are involved in the development and implementation of OnlinePsyHulp, an online platform for mental health problems.

## Data Availability

The data are not freely available due to third-party rights.
